# Providing financial protection in health for low-income populations: a comparison of health financing designs in East Asia

**DOI:** 10.1186/s12939-025-02568-2

**Published:** 2025-07-31

**Authors:** Qian Zhang, Julia Shu-Huah Wang, Alex Jingwei He, Chenhong Peng, Aya Abe, Inhoe Ku, Irene Y.H. Ng, Xi Zhao

**Affiliations:** 1 Department of Social Work and Social Administration, The University of Hong Kong, Hong Kong SAR, China; 2 Department of Social Work, National Taiwan University,; 3 Division of Public Policy, The Hong Kong University of Science and Technology, Hong Kong SAR, China; 4 School of Humanities and Social Sciences, Tokyo Metropolitan University, Tokyo, Japan; 5 Department of Social Welfare, College of Social Sciences, Seoul National University, Seoul, Republic of Korea; 6 Department of Social Work, National University of Singapore, Singapore, Singapore; 7 School of Social Research, Renmin University of China, Beijing, China

**Keywords:** Health financing, Social health insurance, Medical financial assistance, Financial protection, Low-income population, Health financing equity, East Asia

## Abstract

**Background:**

Fighting illness and poverty are intertwined objectives in global development. In recent decades, health financing reforms across many nations have enhanced financial protection for low-income populations and promoted health equity for all citizens. However, prior cross-national comparative studies predominantly focused on examining financing structures or social health insurance (SHI) schemes, neglecting financing schemes targeting the poor, such as medical financial assistance (MFA). This study comparatively explores the design of health financing schemes and financial protection outcomes for low-income populations across six societies in East Asia: mainland China, Hong Kong, Taiwan, Japan, South Korea, and Singapore.

**Methods:**

We assess the design of health financing schemes from the dimensions of income-based eligibility, population coverage, and benefit generosity. Policy information was collected from official websites and policy reports. To compare financial protection outcomes, we derived the data through the “model family approach” and jurisdiction-level statistics and simulated catastrophic health spending of lung cancer for individuals across four income levels: (1) no income; (2) earning minimum wage; (3) earning half the national/regional average wage; and (4) earning the national/regional average wage.

**Results:**

We find that health financing schemes in Taiwan and Hong Kong are generous and inclusive for general populations, while Japan, South Korea, and Singapore’s financing schemes are protective and offer relatively generous benefits for vulnerable groups. In contrast, mainland China provides limited benefits in SHI and MFA schemes. Health financing schemes reduce the financial burden to varying degrees, with Taiwan, Hong Kong, and South Korea providing financial protection for low-income populations to a higher degree, followed by Japan, Singapore, and mainland China. Notably, our findings highlight inequities for individuals earning half the average wage in Singapore, mainland China, and Japan (and to a lesser extent in Taiwan, Hong Kong and Korea), as these groups face higher risks of catastrophic health spending compared to other income groups.

**Conclusions:**

Our findings further the understanding of health financing designs in East Asia. We also provide evidence for governments to enhance financial protection for low-income populations, particularly near-poor groups, to achieve more equitable health financing arrangements.

**Supplementary Information:**

The online version contains supplementary material available at 10.1186/s12939-025-02568-2.

## Introduction

Low-income populations are often trapped in a vicious health-poverty cycle that calls for effective policy interventions. Over the past decades, numerous countries have rolled out healthcare reforms aimed at optimizing health financing systems to promote health equity and provide financial protection for low-income populations. Health financing systems, comprising diverse financing schemes such as tax-financed government schemes (e.g., the UK’s National Health Service, NHS), social health insurance (SHI) schemes, and various types of medical financial assistance (MFA) schemes, play a pivotal role in improving access to healthcare and reducing patients’ financial burden [1].

Although many countries have made impressive strides in reducing individual out-of-pocket expenditures (OOP) by introducing SHI, impoverishment due to OOP persists among low-income populations, even in countries with advanced health systems [2]. Consequently, many countries have developed multiple pro-poor health financing schemes to reduce financial barriers for low-income populations and other vulnerable groups [3, 4]. MFA is a commonly adopted approach. In some countries such as Singapore and China, MFA supplements SHI, assisting eligible low-income patients in paying medical bills not covered by SHI. In other countries such as the US, South Korea, and Cambodia, MFA operates as a parallel financing scheme to the SHI scheme.

Although prior studies have widely compared health financing systems and SHI schemes across countries [5–8] few studies systematically compare the design of pro-poor health financing schemes such as MFA. Targeting the most vulnerable groups in society, pro-poor health financing schemes work in conjunction with other financing schemes to achieve financial protection. However, the varied approaches through which countries configure multiple health financing schemes (e.g., SHI and MFA) to improve financial protection are still understudied.

This study compares health financing schemes (i.e., SHI and MFA) and the financial protection outcome (i.e., catastrophic health spending [CHS]) in six East Asian societies: mainland China, Hong Kong, Taiwan, Japan, South Korea [Korea hereafter], and Singapore. East Asia presents a fertile ground for investigating health financing systems given the distinct features of health systems in this region [9, 10]. Inheriting the British tradition, Hong Kong is a typical society with a tax-financed government scheme (GS). Singapore’s system emphasizes personal responsibility and is co-funded by government subsidies, individual savings, and SHI. The other four societies mainly rely on SHI. Japan, Korea, and Taiwan built their SHI systems on the German Bismarckian model and share similar features in benefit packages and service provision [11]. Mainland China operates a dual-track SHI system (with separate schemes for urban employees and residents/rural populations) that has experienced rapid expansions in coverage and benefits since the launch of its new national healthcare reform in 2009. The MFA schemes in the six societies also exhibit diversity. In mainland China, Hong Kong, Taiwan, and Singapore, low-income populations are protected by both SHI or GS and MFA. In Korea and Japan, MFA schemes operate parallel to SHI systems, meaning MFA beneficiaries are not enrolled in SHI schemes. The six societies encompass a wide variety of health financing arrangements, making an investigation into their systems essential for deriving useful lessons for other Asian countries and beyond to consider directions for future reforms.

In this study we aim to answer two research questions: (1) What are the divergent features of East Asia health financing schemes? (2) What are financial protection outcomes for low-income populations across different income levels under varying health financing designs? We first compare the characteristics of health financing schemes, namely SHI/GS and MFA, across the dimensions of eligibility, coverage, and generosity. Subsequently, we analyze the CHS for low-income individuals of different income levels across the six societies. We compare jurisdiction-level statistics and data collected through a “model family approach” (or a vignette approach). The model family approach involves setting up hypothetical family profiles and calculating the amounts of welfare benefits these families are eligible for, as well as family expenses. It allows us to compare policy outcomes for families or individuals with different profiles across societies.

This study contributes to the literature in three ways. First, it is the first comprehensive attempt to compare health financing policy in East Asia. Unlike prior studies that predominantly focus on comparing SHI schemes, we investigate both SHI/GS and MFA and find notable heterogeneities in policy design. Second, in regions where cross-nationally harmonized survey data are unavailable or suitable health expenditure data across countries are lacking, our study pioneers a novel methodological approach. We compare financial protection outcomes among low-income individual of different income levels using the model family approach. Additionally, prior studies commonly used individual or household OOP when comparing health financial burdens in different health financing settings. However, this aggregated indicator often obscures the costs and benefits of varied health financing schemes. We introduce an alternative approach by manually calculating medical costs and benefits to facilitate more nuanced comparisons. Third, the policy implications of this study can offer invaluable insights for countries grappling with mitigating medical impoverishment to achieve more equitable health financing.

## Literature review

Prior studies of cross-regional health system comparison adopted varying approaches, which can be mainly categorized into descriptive and typological approaches. The former illustrates the characteristics and performance of health systems from different dimensions (e.g., population coverage, funding source, benefit packages, service delivery efficiency, and financial protection) through indicators or indexes, such as the share of government health expenditures in total health expenditures, OOP per capita, CHS, and health insurance coverage [11–15]. The latter approach emphasizes the classification of different health systems. For instance, some studies categorize health financing regimes based on funding structure (i.e., state, societal, private, or mixed model) [16, 17], while others develop health system typologies using cluster analysis based on selected indicators [18–20].

Comparative health system studies in the East Asian context have used both approaches, revealing both commonalities and differences. Regarding similarities, health systems in East Asia all emphasize the government’s regulatory role, even in Korea, Taiwan, and Japan where the private sector dominates service provision [11]. The central government bears the ultimate responsibility for health policy implementation and monitoring. In terms of heterogeneities, East Asian societies have developed different healthcare regimes drawing from British or German experiences, resulting in distinct features in both financing and provision [20]. For instance, the public sector provides most hospital beds in mainland China, Hong Kong, and Singapore, and an opposite landscape is manifested in Taiwan, Japan, and Korea. Hwang [13] notes that health systems in East Asia are geared toward different objectives; for example, mainland China emphasizes the political significance of health policy, while Japan focuses more on social and medical objectives. In addition, some studies compared individual or household outcomes of different health system designs, including OOP, CHS [10, 21], and health service utilization [10, 22]. However, recent research has paid limited attention to comparing financial protection outcomes and MFA in East Asian societies.

Protection against the financial risk of illness stands as a key goal of health financing. While many studies have endorsed SHI or MFA’s positive effects of financial protection in reducing OOP and the occurrence of CHS [23–25], others have found limited or no impact on financial protection [26–28]. The design of MFA benefit packages, including deficiencies in eligibility criteria and service coverage, can explain the non-significant association between MFA and financial burden [29]. Liu et al. [26] also argue that the expansion of SHI boosts insurees’ healthcare utilization and encourages the profit-seeking behaviors of health service providers, potentially offsetting the positive effects of SHI.

Prior studies provide valuable references for comparing and evaluating health financing globally and across East Asia, but they mostly focus on SHI [7, 21], paying limited attention to pro-poor health policies, especially MFA. Furthermore, there is a dearth of comparative knowledge regarding the configuration of SHI/GS and MFA within a health system across countries. SHI/GS and pro-poor schemes exist simultaneously in most countries’ health systems and act in coordination to achieve financial protection. Analyzing both schemes in the health financing design can reveal which configuration pattern achieves better financial protection outcomes for the poor. This provides important evidence for other countries to improve their health financing structure in reducing the financial burden for the poor.

## The East Asian context

Health systems in East Asia exhibit distinct features shaped by diverse socioeconomic structures. In Appendix A, we provide an overview of socioeconomic contexts and health system characteristics in six societies. Below, we review each society’s health system with a focus on financing and provision. Table 1 summarizes key policy information of SHI/GS and MFA.

### Mainland China

China’s health system is primarily financed by two SHI schemes: Urban Employee Basic Medical Insurance (UEBMI) and Urban and Rural Resident Basic Medical Insurance (URRBMI). The former targets urban employed residents, while the latter covers rural residents and urban residents not enrolled in UEBMI, providing coverage for 70% of the population. The copayment rate of UEBMI is generally lower than that of URRBMI, making it more generous for the insured vis-à-vis URRBMI. Healthcare provision is public-dominant, with over 80% of services provided by the public sector.

In 2008, China launched the Medical Financial Assistance (医疗救助) scheme. It complements the SHI schemes, providing cash aid to eligible low-income households after they receive SHI reimbursement. Eligible households primarily include social assistance (低保) recipients, indigent elderly lacking working ability and family support (特困供养人员), and other low-income populations. The SHI and MFA benefit packages are determined by the prefectural authorities. SHI benefit levels differ based on the healthcare institution’s level, typically with lower-level hospitals offering a higher reimbursement rate.

### Japan

Japan is the first Asian country to establish a comprehensive SHI scheme. Structured along occupational lines, Japan’s SHI system involves multiple insurers and traces its origin to the nation’s pursuit of industrialization [30]. The two main SHI schemes are the Employees’ Health Insurance (EHI) and National Health Insurance (NHI). EHI caters for company employees and their dependents, covering over 50% of the total population. NHI targets self-employed and unemployed people and pensioners under 75. While the private sector dominates health service provision, municipal governments retain authority over provision and financing.

SHI benefits are standardized nationally across all schemes, ensuring universal and equitable access to health services [11]. Both EHI and NHI implement monthly copayment ceilings (高額療養費制度), where any household’s combined medical expenditures exceeding the designated limit which determined by income level and age, the system provides reimbursement to reduce the excess burden. Lower-income groups receive full reimbursement while higher-income groups follow a progressive formula to cap out-of-pocket payments. Patients aged 70 or above enjoy lower monthly caps. Japan’s MFA scheme (Public Medical Assistance, 醫療扶助), is an important component of the public assistance system. Beneficiaries are completely exempted from medical costs.

### South Korea

Korea’s health system is financed by National Health Insurance (NHI), with about 97% of the population covered by the scheme, and the remaining 3% are enrolled in MFA. The copayment rate for health services varies depending on the level of health providers. Korea’s NHI also implements an annual copayment ceiling based on income deciles, where co-payment amounts exceeding the designated ceiling during one fiscal year are reimbursed by National Health Insurance Service. Notably, registered cancer patients benefit from a reduced copayment rate of only 5%. Korea’s healthcare provision relies heavily on private providers, though some public health facilities provide necessary services at the central, regional, and municipal levels [31].

Korea’s MFA scheme, also known as the Medical Aid Scheme (의료급여), is an integral part of the National Basic Livelihood Security Program (BLSP). Eligible populations include BLSP beneficiaries such as those with no work ability, individuals registered as patients with rare and incurable diseases, and other vulnerable populations (e.g., disaster victims, adopted children aged 18 or below). Generally, eligible populations should meet the criteria that they are without an obligated provider or that their obligated provider is practically unable to provide support, and that their income is below 40% of the standard median income. Differential low-level copayments are applied for insured medical services based on beneficiary type.

### Taiwan

Taiwan achieved universal health coverage in 1995 after consolidating various separate insurance schemes into the single-payer National Health Insurance (NHI, 全民健保). Taiwan’s NHI is hailed as a “miracle”, due to its extensive benefit package and minimal user fees paid by patients. Similar to Japan and Korea, the private sector dominates most healthcare delivery in Taiwan, with private hospitals comprising approximately 90% of all hospitals and providing 65% of hospital beds. Taiwan’s NHI implements catastrophic illness coverage, where patients with certified catastrophic illnesses (重大傷病) are completely exempt from copayments for medical services related to their certified condition. This program covers approximately 30 categories of catastrophic diseases and conditions. In addition, Taiwan’s system provides copayment waivers for certain vulnerable groups, such as people aged 100 or above, children under three years old, and those covered by labor insurance for work-related injuries.

Low-income individuals whose household income per capita is below the local low-income or middle-to-low-income minimum living standard (MLS) are eligible for the MFA scheme (Medical subsidies, 醫療輔助). For those below the MLS, copayments are waived entirely, while middle-to-low-income individuals (1.5 times the MLS) receive 80% copayment waivers with a ceiling of assistance. In addition, Taiwan provides special MFA to lower income older adults (below 2.5 times the MLS). The assistance rate varies by city and county, typically ranging from 50 to 70%.

### Singapore

Singapore’s health system is characterized by limited risk pooling, rooted in the political philosophy of “no free lunch,” which encourages personal responsibility for welfare [32]. Health financing primarily relies on government subsidies and the “3 M Model”: Medisave, Medishield Life, and Medifund. Medisave is a compulsory personal savings account where individuals save 6-8% of their monthly income to cover health expenses [20]. Medishield Life is a compulsory SHI scheme to offset high inpatient costs, while deductibles and unclaimable expenses are paid through Medisave or cash. Outpatient costs are mostly paid out of pocket, with selected outpatient treatments paid by Medisave. The public sector plays a substantial role in healthcare provision.

Medifund was introduced to cover medical expenses of vulnerable groups. Patients encountering financial difficulties after exhausting all other avenues such as Medishield Life and Medisave balances, can apply for complete or partial waivers once they pass a means test. Applications undergo assessments on a case-by-case basis, considering families’ financial, health, and social circumstances. Medifund Silver and Medifund Junior are carved out from Medifund to provide more targeted assistance for the needy older adults and children respectively. Generally, the benefits are more generous for these populations. For instance, Medifund Silver provides full subsidies in some cases for older adults in need.

### Hong Kong

Hong Kong adopted the tax-financed health system modeled after the UK’s NHS system [14]. Unlike its residualist approach to welfare, the government actively intervenes to a significant degree in healthcare [33]. Inpatient services are predominantly public, with about 95% of hospitalization provided by the public sector, while 70% of outpatient services are delivered privately. The public sector provides highly subsidized health services, with patients making minimal copayments. However, the long waiting time for public outpatient care is a persistent challenge in the system. The private sector provides more accessible and efficient services for those who are willing to pay more.

Hong Kong also implemented the MFA scheme (Mechanism of Waiving of Medical Charge, 醫療費用減免機制) for vulnerable groups, such as recipients of Comprehensive Social Security Assistance (CSSA, Hong Kong’s last-resort safety net), eligible old adults (i.e., Level 0 voucher holders of the Residential Care Service Voucher Scheme for the Elderly and Old Age Living Allowance recipients aged 75 or above), and other vulnerable patients in need. Beneficiaries are exempted from paying public medical fees.

**Table 1:** Policy information of SHI/GS and MFA in six societies (2020)

		Mainland China (Beijing)	Hong Kong	Taiwan (Taipei)	Japan (Tokyo)	South Korea (Seoul)	Singapore
**SHI/GS**	**Program**	**UEBMI / URRBMI**	**GS**	**NHI**	**EHI / NHI**	**NHI**	**Medishield Life**
	Copayment rate						
	*Outpatient*	10-20% / 45-50%^a^	10-20%^e^	6%^g^	10-30%^l^	30-60%^p^	10%^u^
	*Inpatient*	0.9-15% / 20-22%^a^	3–5%^e^	5-30%^h^	10-30%^l^	5-20% ^p^	3-10%^u^
	*Severe illness*	NA	NA	0^i^	NA	cancer: 5%, rare/incurable disease:10%^p^	NA
	Deductibles	Yes^a^	NA	No^h^	No^l^	No^p^	Yes^u^
	Limit of SHI claims	Yes^a^	NA	No^h^	No^l^	No^p^	Yes^u^
	Limit of copayment	No	No	No	Yes^m^	Yes^p^	No
**MFA**	**Program**	**Medical Assistance**	**Mechanism of Waiving of Medical Charges**	**Medical subsidies**	**Public Medical Assistance**	**Medical Aid**	**Medifund**
	Target population	Social assistance recipients and other vulnerable groups	CSSA recipients, the eligible elderly, and other vulnerable groups^f^	Low- and middle-to- low-income people and other vulnerable groups	Public assistance recipients and other vulnerable groups	BLSP recipients and other vulnerable groups^q^	Patients facing financial difficulties
	Income-based eligibility criteria	Household income per capita is below local minimum livelihood standard or low-income line^b^	Household income does not exceed 75% of the Median Monthly Domestic Household Income applicable to household size^f^	Household income per capita is below the local low-income line or middle-to-low-income line^j^	Household income is below local livelihood assistance standard^n^	Household income is below 40% of the median income^r^	Scrutinization on a case-by-case basis^v^
	Eligibility threshold	2,200 Yuan^c^	7,125 HKD^f^	24,293 NTD^k^	17,4236 Yen^o^	702,878 Won ^s^	650 SGD^w^
	Cash assistance rate						
	*Outpatient*	80-100%^d^	100%^e^	80-100%^j^	100%^n^	500 to 2,000 KRW copayments^t^	Scrutinization on a case-by-case basis^v^
	*Inpatient*	80-100%^d^	100%^f^	80-100%^j^	100%^n^	90-100%^t^	
	*Severe illness*	85-100%^d^	100%^f^	NA	100%^n^	100%^t^	
	Deductibles	No^d^	No^f^	Conditional^j^	No^n^	No^t^	
	Limit of assistance amounts	Yes^d^	No^f^	Conditional^j^	No^n^	No^t^	

Notes

All local currency units were converted to USD units using 2020 PPP exchange rates and consumer price indices (CPIs)

The eligibility threshold refers to the income-based threshold for an individual/single adult family. We take the lowest threshold that represents the most lenient scenario in each society

Data sources: refer to Appendix D1

## Analytical approach

We examine health financing policy features through eligibility, coverage, and generosity. Further, to assess the financial protection outcome, we utilize CHS to explore the extent to which medical costs result in financial burden on individuals. The design of health financing systems is complex, and countries face trade-offs between generous coverage and fiscal affordability [34]. With fixed social welfare funding, broader coverage might lead to lower levels of generosity, and vice versa [18]. In this study, eligibility refers to income-based criteria that determine whether the poor are eligible for benefits [35]. Coverage represents the population covered by welfare programs. Generosity refers to the level of welfare benefits provided to beneficiaries [36]. CHS is a commonly used indicator in health financing evaluation, measuring households’ OOP relative to a predetermined threshold of household capacity-to-pay [37].

## Methodology

### Data collection

To investigate policy features of financing schemes and the financial protection outcome among low-income populations, we collect both policy indicators and simulate individual-level data in six societies. Policy indicators (i.e., eligibility, coverage, and generosity) of SHI/GS and MFA schemes are collected from official websites and policy reports. When the information is unavailable, we consult government officials, experts, or health professionals in the respective jurisdiction.

Regarding financial protection outcome, we use the model family approach to simulate this information based on individuals of different pre-specified income levels. The model family approach captures how a policy works in hypothetical family situations and thereby facilitates cross-country comparison of social welfare systems [38]. Researchers collect information on labor income, tax and social contributions, and cash benefits that a family typically pays and receives and calculate family disposable income and amounts of benefit [39]. Prior studies commonly use the model family approach in comparative policy research [39–43].

We collect model family data based on the single adult (aged 45) situation, capturing family income and expenses, for four hypothetical income levels: (1) no income; (2) earning minimum wage in each society (part-time, working 16 h per week); (3) earning half the national average wage; and (4) earning the national average wage. We opt to collect individual-level rather than family-level data for simplicity. As severe illness is more likely to result in a heavy financial burden, we simulate a scenario whereby individuals face a catastrophic health event. We assume all individuals have lung cancer, because it is the most common cancer in East Asian societies [44].

Medical costs for individuals facing lung cancer situation are captured by medical copayments of lung cancer.^1^ We multiplied the average days of lung cancer hospitalization by the average cost per hospitalization day in each society. This estimation method is drawn from an earlier study investigating the economic burden of cancer across the European Union [45]. This approach enables the comparison of lung cancer burden between countries. We calculate and collect the average cost per outpatient visit/hospitalization day, average health care utilization per person (e.g., average outpatient visits per year), individual monthly income (e.g., wage and cash transfers), tax, social security contributions, consumption (e.g., costs for tax, food, housing, transportation, utilities) for each individual based on government statistics or official reports. The information is shown in Appendix Table A1.

Given subnational variations in policy design in a society, we focus on collecting data for the largest/capital city in each society: Beijing, Hong Kong, Taipei, Tokyo, Seoul, and Singapore. As these cities are the most affluent in each society and have more generous welfare provision, our comparison is presumably based on the most generous situation. Besides, we collect our data based on the following assumptions. First, we assume that no individual holds any assets that would disqualify them from receiving MFA benefits. Second, we assume that all health services utilized by individuals are within the benefit scope of the SHI and MFA schemes in each society, excluding self-funded health services such as cosmetic surgery.

### Measurement

#### Policy design

##### Eligibility and coverage

As SHI/GS is designed to cover all populations, we only focus on the eligibility and coverage of MFA schemes. The eligibility of MFA is measured by income-based eligibility thresholds as a percentage of the national/regional average wage. A higher eligibility rate indicates the policy design tends to enroll more people in the MFA scheme. Coverage is measured by total MFA recipients as the percentage of the total population in a society.

##### Generosity

Generosity captures the benefit levels patients can receive under SHI/GS and MFA. SHI/GS generosity is measured by medical copayments as the percentage of the national average wage. We estimate outpatient copayment per visit, inpatient copayment per attendance, and total copayment for each lung cancer case. Under Hong Kong’s GS system, we used fixed public charges as patients’ copayments. MFA generosity is measured using the assistance rate, which represents the total MFA benefits received by beneficiaries as a percentage of total copayments for non-beneficiaries. For instance, if beneficiaries do not need to pay any medical costs, the assistance rate is 100%.

#### Financial protection outcome

##### Catastrophic health spending

CHS refers to households’ medical costs relative to a predetermined threshold of household capacity to pay [37]. We measure the capacity to pay by individual total disposable income (total income hereafter).^2^ If medical costs exceed 10% of total income, individuals are considered to be facing CHS [2]. In Singapore, the amount of Medifund benefits distributed to the beneficiaries largely depends on patients’ family financial circumstances (e.g., income and assets) and Medisave balances. Given the absence of official statistics on average Medisave balances, we compute medical costs using the following assumptions: (1) Individuals with no wage lack sufficient Medisave balance, hence receiving full Medifund assistance; (2) For individuals earning minimum wage, we estimate two scenarios: (2.1) those with sufficient Medisave receive no Medifund support, and (2.2) those without sufficient Medisave receive full Medifund assistance. In mainland China, we assume individuals having no wage are enrolled in URRBMI, while individuals of other income cases participate in UEBMI.

We also conduct robustness checks. First, to address potential concerns about the representativeness of the lung cancer case, we simulate a more common healthcare scenario where all individuals experience the national/regional average outpatient utilization rate. We estimated the outpatient financial burden in each society by multiplying the average outpatient costs per visit by the average number of outpatient visits. Second, we use two alternative measurements of capacity to pay. Third, we adjust for variations in utilization or treatment practice across societies by estimating costs based on the average length of stay of lung cancer in OECD and EU countries.

### Empirical strategies

In this study, we conduct descriptive analyses to answer research questions. We explore the policy features of SHI/GS and MFA based on eligibility, coverage, and generosity. Next, we compare the CHS of individuals with different income levels across six societies. STATA 16.0 is used for data analysis.

## Results

### Policy design

#### Eligibility and coverage

As shown in Table 2, income-based eligibility for MFA is the most lenient in Japan, followed by Taiwan. Paradoxically, the two societies perform poorly in coverage, only covering around 2% of the population. Singapore provides the strictest income-based eligibility for MFA enrollment, while ranking second in population coverage. Similarly, Hong Kong ranked third in eligibility leniency, while ranking top in population coverage. Korea performs averagely in eligibility and coverage, while mainland China provides moderately lenient eligibility but has the lowest coverage.

This discrepancy in income-based eligibility and coverage shows that eligibility criteria aside from income thresholds affect coverage, such as household assets, age, or illness. For instance, Japan’s means test is fairly strict, considering all available resources, including household assets, ability to work, and support from legally obligated individuals, which might result in low population coverage [46]. In contrast, MFA in Hong Kong adopts categorical eligibility criteria. In addition to recipients of CSSA, it also targets other vulnerable groups such as chronically ill patients and old patients who have little income or assets. Despite not having the most lenient income-based eligibility, it provides the highest population coverage.

#### Generosity

Outpatient copayments relative to the national average wage are the highest in mainland China, and the lowest in Taiwan. In terms of inpatient services, Singapore charges relatively higher copayments, followed by Japan, Korea, and mainland China. Regarding lung cancer, Taiwan’s SHI scheme is the most generous, exempting patients from copayments regardless of individuals’ income situation, while in Singapore and mainland China, SHI schemes’ coverage of medical bills for lung cancer patients is limited.

For MFA, Hong Kong, Taiwan, Japan, and Singapore all provide 100% assistance for eligible low-income patients. However, in mainland China and Korea, eligible patients still have to pay small copayments after MFA benefits. Regarding lung cancer, most societies offer full assistance to patients, except for mainland China where patients might still incur some costs despite receiving benefits.

**Table 2:** Policy features of SHI/GS and MFA

			Mainland China (Beijing)	Hong Kong	Taiwan (Taipei)	Japan (Tokyo)	South Korea (Seoul)	Singapore
**Income-based Eligibility**	MFA	Income-based threshold as % of the national/regional average wage	27.1%	37%	44.7%	56.6%	22.1%	14.3%
**Coverage**	MFA	Recipients as % of the total population	1.06%^a^	20.51%^d^	2.6%^h^	1.63%^l^	2.95%^p^	9.7%^t^
**Generosity**	SHI/GS	Outpatient copayment per visit, US$	47.14^b^	18.44^e^	7.66^i^	28.74^m^	23.42^q^	38.54^u^
		Outpatient copayment per visit as % of the national/regional average wage	2.4%	0.6%	0.2%	1.0%	0.6%	0.7%
		Inpatient copayments per attendance, US$	566.36^b^	170.54^e^	411.92^i^	970.30^m^	1,152.734^q^	1,860.25^v^
		Inpatient copayment per attendance as % of the national/regional average wage	29.2%	5.4%	10.5%	32.4%	31.2%	34.4%
		Average hospital length of stay for lung cancer, days	11.80^b^	9.2^f^	9.6 ^j^	11.64^n^	13.7^r^	12.9^w^
		Lung cancer copayments per case, US$	681.31	196.12	0.00	560.12	188.53	2,280.89
		Lung cancer copayment per case as % of the national/regional average wage	35.1%	6.2%	0%	18.7%	5.1%	42.2%
	MFA	Outpatient assistance rate	80%^c^	100%^g^	100%^k^	100%^o^	90%^s^	100%^x^
		Inpatient assistance rate	80%^c^	100%^g^	100%^k^	100%^o^	88%^s^	100%^x^
		Lung cancer assistance rate	85%^c^	100%^g^	NA	100%^o^	100%^s^	100%^x^

Notes

All local currency units are converted to USD units using 2020 PPP exchange rates and consumer price indices (CPIs)

To ensure consistency, we use the lowest eligibility threshold if it varies in different vulnerable groups in a society. Similarly, we use the highest assistance rate if different levels of assistance rates exist. Therefore, we compare the most generous situation in six societies. We calculated SHI generosity based on benefits received by individuals earning the average wage

Lung cancer copayment per case is estimated by the average length of stay of lung cancer multiplied by the average inpatient copayment per day in each society

In Japan, the calculated lung cancer copayment (77,052 yen) is higher than the copayment ceiling (57,600 yen, US$560.12) for individuals earning the national average wage, so we applied this ceiling. In Taiwan, copayment for severe illness is completely waived, and in Korea, the copayment rate for severe illness is 5%

Data sources: refer to Appendix D2

**Fig. 1:**
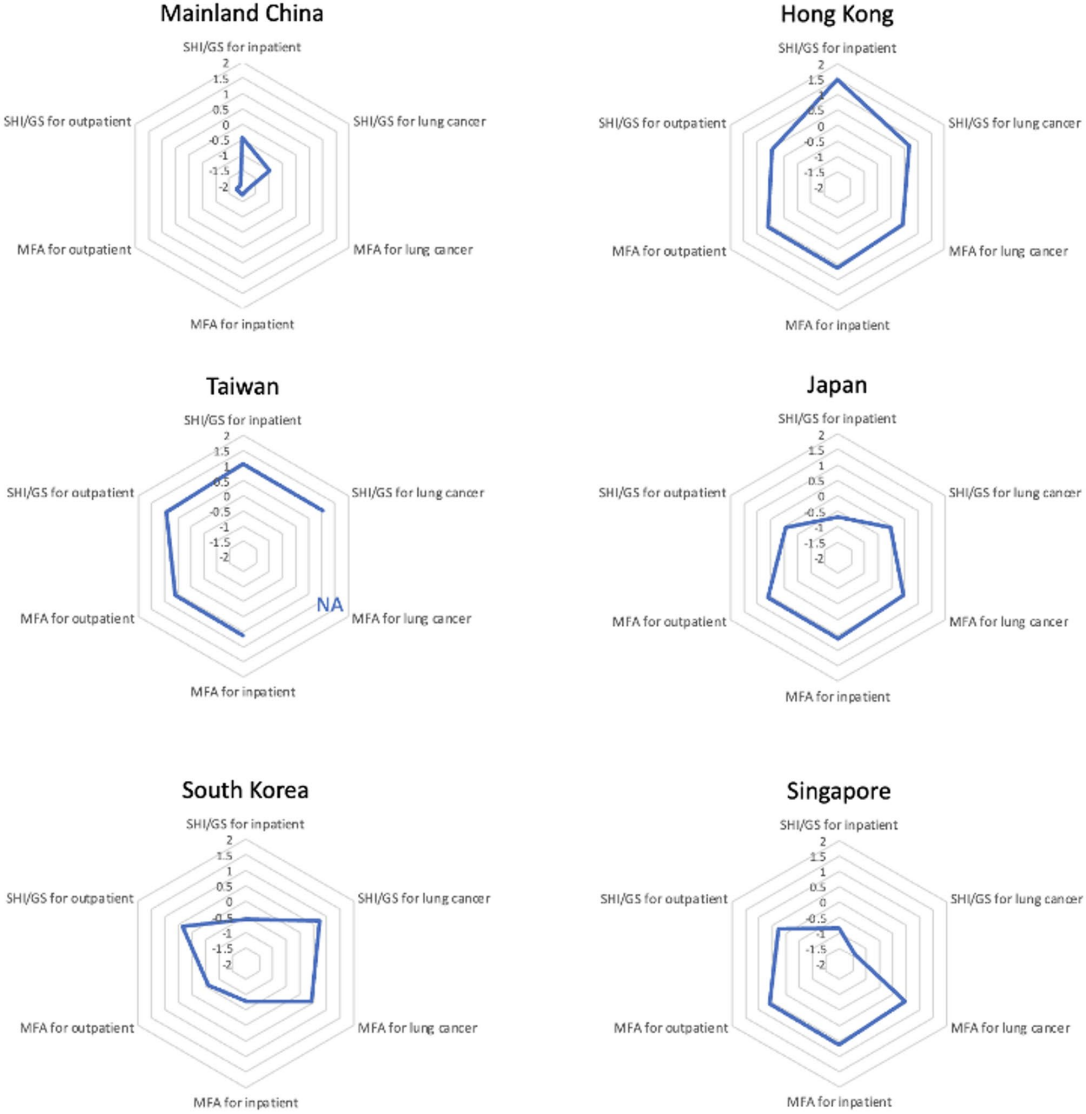
Generosity patterns of health financing schemes

In Fig. 1, we summarize SHI/GS and MFA generosity patterns in each society based on standardized scores, which offers insight into the relative position of each society’s level of generosity compared to others. Taiwan and Hong Kong provide generous benefits for all income cases, with SHI/GS and MFA benefits being particularly high for inpatient, outpatient, and lung cancer cases. In comparison, Japan and Singapore provide moderate and low SHI benefits, respectively, but offer adequate MFA generosity that benefits the poor. Korea offers a higher level of benefits for individuals with cancer, while mainland China provides comparatively lower SHI benefits and the least MFA benefits.

### Financial protection outcome

**Fig. 2:**
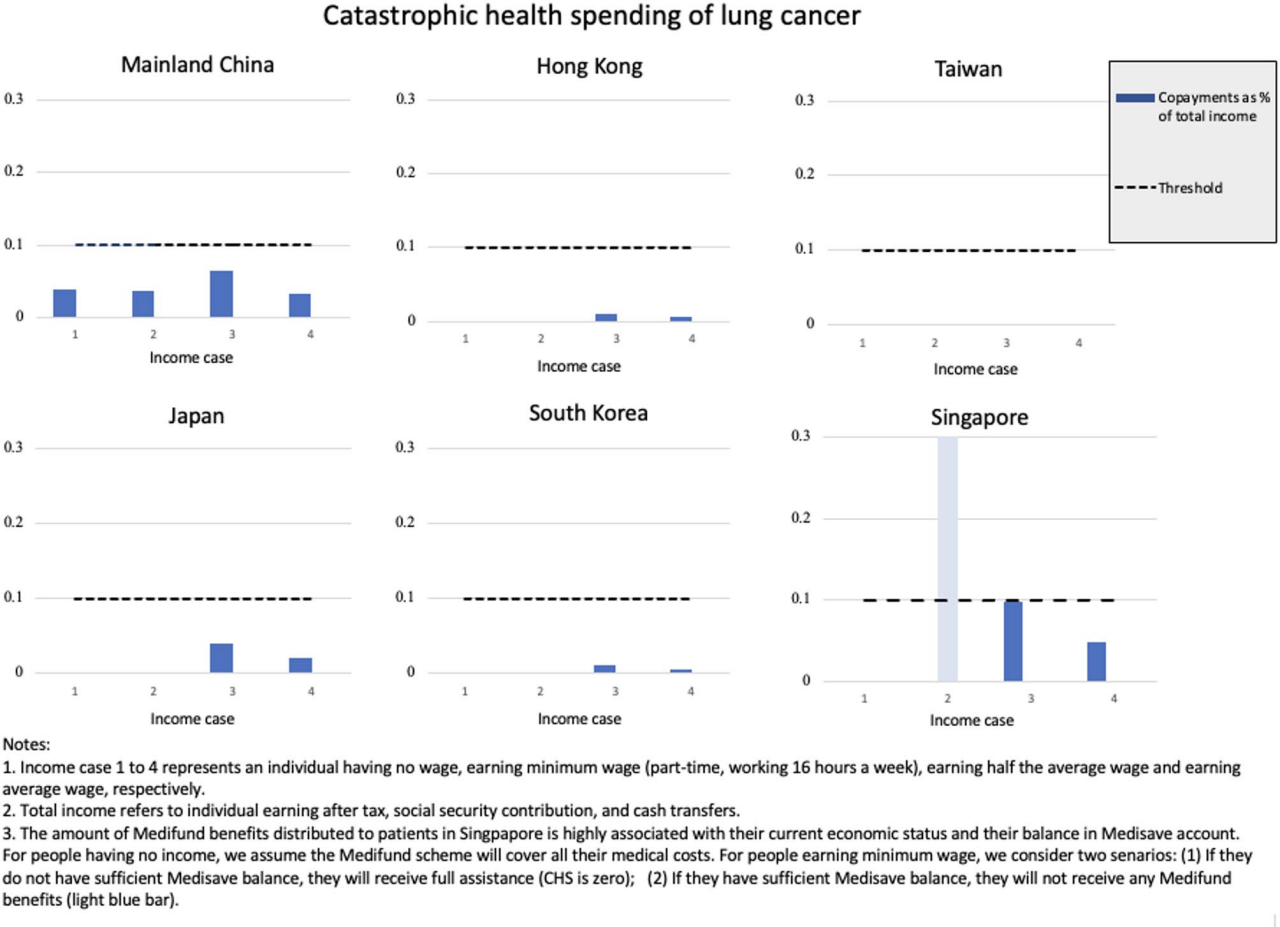
Catastrophic health spending of lung cancer

Figure 2 shows the results of CHS of lung cancer across six societies by the four income profiles. In Taiwan, where lung cancer costs are fully covered by the SHI scheme, copayments relative to total income are zero across all income cases. Hong Kong and Korea exhibit similar patterns, with figures nearing zero for all income cases. These findings underscore the robust protection provided by health financing schemes in Taiwan, Hong Kong, and Korea for low-income populations facing severe illness.

For individuals having no wage or earning minimum wage, copayments relative to total income are zero in Japan and below 5% in mainland China. This indicates that the two societies shield low-income populations from CHS. Similarly, in Singapore, MFA effectively protects individuals with no wage or earning minimum wage, provided that they lack sufficient Medisave balance. Nevertheless, individuals earning minimum wage with sufficient Medisave balance may still encounter CHS after exhausting all their benefits. However, for most Singaporeans, these medical expenses are unlikely to significantly impact their lives, as they can be deducted from their Medisave balance.

Furthermore, our analysis reveals that, after MFA benefits, individuals earning half the national average wage in Singapore, mainland China, and Japan are more likely to incur CHS. Their copayments relative to total income are much higher than other income groups. This heightened their vulnerability to catastrophic health events due to the absence of protection from MFA benefits or their income.

**Fig. 3:**
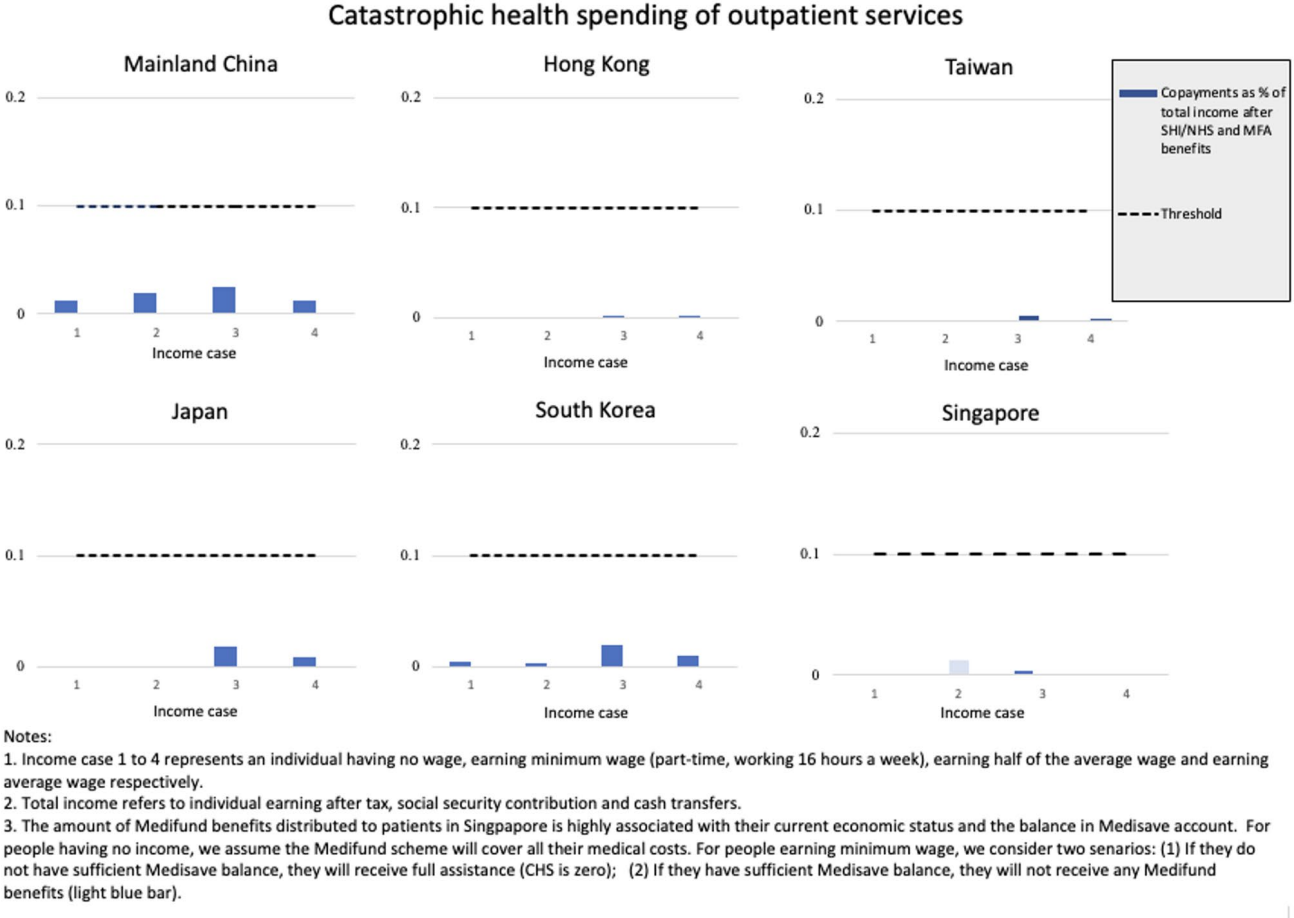
Catastrophic health spending of outpatient services

For robustness check, we also simulate a common healthcare scenario where individuals experience the national/regional average outpatient utilization rate. This scenario better captures the financial protection level for routine healthcare needs across different diseases. Figure 3 shows the results. Average outpatient services do not cause CHS for any individuals across all income levels. Taiwan and Hong Kong show consistently generous protection for all income categories. Compared with lung cancer scenario, Korea shows less protection for people under average healthcare utilization scenarios, while in Singapore’s system, people under average scenarios have very minimal medical burden. Japan and mainland China exhibit similar patterns to those observed in the lung cancer scenario. Individuals earning half the national average wage remain at risk of CHS in all societies. The results of additional robustness checks (including alternative measurements of capacity to pay and analyses based on OECD and EU countries’ situations) are presented in Appendix B.

## Discussion

This study compares the design of health financing schemes and financial protection for low-income individuals in six distinct East Asian health systems. These systems are financed by tax (Hong Kong), SHI (mainland China, Taiwan, Japan, and Korea), and co-financed by government subsidies, individual savings, and SHI (Singapore). We provide a summary of overall findings in Table 3 and classify six societies into different health financing regimes.

**Table 3:** Features of health financing designs in six societies

	Mainland China	Hong Kong	Taiwan	Japan	South Korea	Singapore
MFA eligibility	Moderate	Lenient	Lenient	Most lenient	Moderate	Stringent
MFA coverage	Low	Highest	Moderate	Low	Moderate	High
SHI/GS generosity	Low	High	Highest	Moderate	Moderate	Low
MFA generosity	Low	High	High	High	Moderate	High
Financial protection	Moderate	High	Highest	High	High	Moderate
Regime classification	Minimalist model	Inclusive model	Inclusive model	Protective model	Protective model	Protective model

Taiwan and Hong Kong are both classified as the *inclusive model*, offering generous benefits to all citizens. Taiwan offers the most generous benefit levels among the six societies. Notably, Taiwan’s SHI offers a more comprehensive benefit package, including low copayments and high-covered services, compared to other systems [21]. Similarly, Hong Kong provides generous benefits to all citizens, with the government heavily subsidizing public health services. Moreover, regarding MFA schemes, both societies set lenient income-based eligibility criteria and provide moderate to high coverage. These findings align with previous research indicating a lower incidence of CHS in Taiwan and Hong Kong, contrasting with other East Asia societies [2, 10, 21]. However, despite Hong Kong’s classification as an *inclusive model*, its low-cost public healthcare services are less accessible due to significantly prolonged waiting time. We can find Hong Kong has the highest OOP per capita (Appendix A), primarily due to expenses incurred in the private sector. Also, the ultra-generous SHI system in Taiwan faces financial sustainability challenges [47].

Japan, Korea, and Singapore are all categorized under the *protective model*. Japan and Singapore provide generous MFA benefits targeting low-income populations, which surpasses their relative performances in SHI schemes. Korea demonstrates particularly generous benefits for cancer patients. From 2004 to 2009, Korea consistently reduced the copayment for cancer patients, decreasing it from 20 to 5% [48]. This expanded benefit leads to a reduced financial burden for cancer patients across all income groups when compared to those with other diseases [49]. Regarding CHS when facing lung cancer in Japan, lower-income individuals receive better protection compared to their wealthier counterparts. This can be explained by the generous MFA scheme in Japan that waives insurance premium costs and copayments for qualifying individuals. Similarly, Singapore provides robust protection for the poorest group (i.e., those with no income). However, Singapore stands out in that higher-income groups still face substantial financial burdens, especially for those experiencing severe diseases such as lung cancer. The strong emphasis on individual responsibility in Singapore arguably sustains a healthcare system with high OOP for non-poor groups [50]. Paradoxically, despite the low healthcare spending, Singapore is one of the healthiest nations, leading some to argue that it is an exemplar of achieving the most efficient health system by maintaining a delicate balance between efficiency and equity [32].

Mainland China is classified as a *minimalist model* with comparatively lower generosity in both SHI and MFA among six societies. While its health financing schemes offer protection for low-income populations, keeping CHS below the 10% threshold, the level of protection is still relatively insufficient compared to other societies. Since the launch of health system reform in 2009, significant progress has been made in expanding the benefits and population coverage of both SHI and MFA in mainland China. As a result, the incidence of CHS has shown a notable downward trend [51]. However, there remains ample room for expansion in benefit packages for vulnerable individuals, considering income-related, disease-related, and regional disparities [51, 52]. While mainland China is making concerted efforts to catch up with other peers in terms of benefit levels, there is a substantial journey ahead.

Furthermore, a noteworthy finding emerges in Singapore, mainland China, and Japan (and to a minimal extent in Taiwan, Hong Kong and Korea), where individuals earning lower wages but not yet classified as poor (e.g., earning half the average wage) are disproportionately susceptible to incurring CHS compared to other lower or higher income groups. These marginalized populations lack the support of adequate earnings or welfare benefits. For example, in Singapore, those earning half the average wage are not poor enough to qualify for Medifund. Their CHS of lung cancer reaches the critical threshold of 10%. This trend across many societies underscores the urgent need for enhanced protection for lower-income populations that are ineligible for MFA.

While calling for attention to meet the needs of near-poor populations, we must also acknowledge competing macro-level challenges such as demographic shifts and economic recessions that threaten the financial sustainability of generous health financing systems. In aging societies like Japan, labor shortages coupled with increased healthcare demand inevitably strain healthcare systems that mainly rely on employment contributions [53]. Economic recessions further cause unemployment, leaving families in vulnerable positions needing more services and assistance, while simultaneously reducing tax contribution bases to finance these services [54]. Prior literature has argued that health financing systems relying predominantly on employment contributions are unsustainable, and it is necessary to diversify financing sources and increase tax revenues allocated to the health sector [53, 55]. Each society must deliberate on how to acquire and manage financial resources to enhance financing efficiency and strike a balance between generous benefits and financial constraints.

In summary, the inclusive model, characterized by high SHI/GS and MFA benefits, achieves the strongest financial protection for all citizens. The protective model, featuring moderate or low SHI/GS benefits but high MFA benefits, tends to provide optimal financial protection for economically vulnerable groups. Finally, the minimalist model, with low SHI/GS and MFA benefits, provides relatively limited financial protection, resulting in individuals with lower incomes still facing excessive health financial burdens compared with other models. The latter two models are more susceptible to exhibiting disparities between welfare beneficiaries and marginalized groups ineligible for MFA. Expanding the coverage of MFA benefits alongside enhancing generosity could improve financial protection for lower-income populations.

This study has several limitations. First, we use a simplified method to calculate CHS of lung cancer. While this approach allows for the comparability of estimation across countries, it unavoidably underestimates the actual financial burden by excluding additional costs such as outpatient, transportation, and meal costs. Second, we only collected data from the public sector due to a lack of comprehensive access to data covering private healthcare. This underestimates medical costs, particularly in Hong Kong, where the unsubsidized private sector delivers most outpatient services. Third, our calculation of medical costs is based on the national/regional average level of unit medical costs and healthcare utilization, which fails to capture variation across different income types. For example, lower-income individuals may have less healthcare utilization than wealthier populations [56]. Additionally, this study focuses on individual scenarios and does not capture the complex health needs of households, such as children or the older adults who may have more medical need. Furthermore, our focus on capital cities does not reflect regional variation within societies. Fourth, this study is based on data from 2020, which raises questions about the potential influence of the COVID-19 pandemic on the results. We find that all societies had relatively stable SHI and MFA designs that did not change significantly during the pandemic. Furthermore, healthcare utilization decreased across all societies after the outbreak (see Appendix C), and the relative ranking of healthcare utilization remained similar to pre-pandemic levels. Therefore, we conclude that the results still reflect relative performance across societies. Fifth, due to sample size limitations, we are unable to conduct statistical analysis such as significance test, which could facilitate better comparisons. Future studies could either use alternative data sources or, if applying the model family approach, include a wider range of individual or family profiles to allow for more comprehensive comparisons. Finally, although our findings indicate effective financial protection for low-income populations in six societies, we only examine the ideal policy situation where eligible populations indeed benefit from the program. The actual policy implementation may result in varying extents of benefits for low-income populations.

## Conclusions

This study reveals the heterogeneous health financing regimes in East Asia: *inclusive model* (Taiwan and Hong Kong), *protective model* (Japan, Korea and Singapore) and *minimalist model* (mainland China). Comparatively, inclusive model is more equitable in health financing, providing the most generous benefits and strongest financial protection for all income groups. The latter two models are more susceptible to exhibiting inequality in benefits and financial outcomes among different income groups. The results suggest that individuals earning lower wages but not yet classified as poor face higher risk of incurring catastrophic health spending compared to other income groups. This study represents a new attempt to comparatively investigate health financing for low-income populations across societies and may offer valuable insights for health financing design.

## Electronic supplementary material

Below is the link to the electronic supplementary material.

Supplementary Material 1

Supplementary Material 2

## Data Availability

The datasets used and/or analysed during the current study are available from the corresponding author on reasonable request.

## Footnotes

1 Medical copayments are the portion of cost-sharing that is paid out of patients’ pockets under SHI schemes.

2 In this study, individual total income refers to individual earning after tax, social security contributions, and cash transfers.

## Abbreviations

SHI: Social health insurance
MFA: Medical financial assistance
NHS: National Health Service
OOP: Out-of-pocket expenditures
CHS: Catastrophic health spending
GS: Government scheme
UEBMI: Urban Employee Basic Medical Insurance
URRBMI: Urban and Rural Resident Basic Medical Insurance
EHI: Employees’ Health Insurance
NHI: National Health Insurance
BLSP: National Basic Livelihood Security Program
CSSA: Comprehensive Social Security Assistance
