# Supplementary material for: Providing financial protection in health for low-income populations: a comparison of health financing designs in East Asia
Source: Int J Equity Health. 2025 Jul 31;24:215. doi: 10.1186/s12939-025-02568-2 (PMC12315319; doi:10.1186/s12939-025-02568-2)
Supplement: Supplementary file 2 — Supplementary Material 2 [file 12939_2025_2568_MOESM2_ESM.doc]

**Appendix A**

**Table A1.** Economic and social contexts and health system characteristics in six societies in 2020 or the most recent year

| Society | | Mainland China | Hong Kong | Taiwan | Japan | South Korea | Singapore |
| --- | --- | --- | --- | --- | --- | --- | --- |
| Economic and social context | GDP per capita, current US$^a^ | 10,525 | 46,443.58 | 28,404.72 | 39,981.48 | 31,728.3 | 60,727.62 |
|  | Monthly average wage, US$ | 1,939.05^b^ | 3,171.86^c^ | 3,913.30^d^ | 2,992.17^e^ | 3,500.72^f^ | 5,410.50^g^ |
|  | Tax and social security contribution as % of national average wage | 10.52% | 6.41% | 5.16% | 22.31% | 20.23% | 28% |
|  | Population (in 10,000) ^h^ | 1 411 10 | 7 48 | 2 356 | 12 626 | 5 184 | 569 |
|  | % elderly population aged 65+^i^ | 14.11% | 20.94% | 18.07% | 29.22% | 18.38% | 13.7% |
|  | Life expectancy at birth, years^i^ | 78.23 | 86.63 | 84.54 | 88.06 | 86.42 | 89.34 |
|  | Under-5 mortality rate per 1,000 live births ^j^ | 10.9 | 2.0 | 4.4 | 2.4 | 2.5 | 1.9 |
| Health system characteristics | Current health expenditures as % of GDP | 5.59% ^k^ | 6.5%^l^ | 6.11%^m^ | 10.9% ^k^ | 8.36%^k^ | 6.05%^k^ |
|  | Out-of-pocket expenditure as % of current health expenditures | 34.79% ^k^ | 29.6% ^l^ | 34.02% ^m^ | 12.57% ^k^ | 27.75%^k^ | 18.97%^k^ |
|  | Out-of-pocket expenditures per capita, current US$ | 202.98^k^ | 923.07^l^ | 599.27^m^ | 551.55^k^ | 733.26^k^ | 670.87^k^ |
|  | Hospital beds per 1,000 population | 4.3^k^ | 4.8^n^ | 7.3^o^ | 13^k^ | 12.4^k^ | 2.5^k^ |
|  | Doctors per 1,000 persons | 2.2^k^ | 2.1^n^ | 2.2^o^ | 2.5^k^ | 2.5^k^ | 2.5^k^ |
|  | Nursing and midwifery personnel per 1,000 persons | 3.1^k^ | 8.3^n^ | 5.9^o^ | 11.9^k^ | 8.2^k^ | 6.2^k^ |
|  | Outpatient visits per capita | 5.5^p^ | 2.35^q^ | 14.44^r^ | 8.88^s^ | 15.41^t^ | 2.18^u^ |
|  | Hospital length of stay, days | 8.50^p^ | 8.00^q^ | 9.35^r^ | 14.5^s^ | 20.9^t^ | 5.24^v^ |
|  | Year of universal health coverage | 2011 | 1964 | 1995 | 1961 | 1989 | 1983 |

Notes.

1. All local currency units are converted to USD units using 2020 PPP exchange rates and consumer price indices (CPIs).
2. Tax and social security contribution as % of national average wage are calculated based on individuals earning the national average wage in the model family data.
3. According to the definition of the World Bank, ‘doctors’ refers to generalist and specialist medical practitioners, and doctors of Chinese medicine are excluded.
4. Outpatient visits involve attendances to all kinds of health institutions in a society, such as emergency attendances, dental attendances, and visits to Chinese medical clinics if applicable.
5. Data sources.

^a^ International Monetary Fund Data Mapper. (2020). GDP per capita (current USD) in mainland China, Japan, Korea, Taiwan, Hong Kong, and Singapore. <https://www.imf.org/external/datamapper/NGDPDPC@WEO/CHN/HKG/JPN/KOR/SGP/TWN>

^b^ National Bureau of Statistics. The average wage of employed persons in urban areas in 2020. <https://data.stats.gov.cn/search.htm?s=%E5%B7%A5%E8%B5%84>

^c^ The Census and Statistics Department. Quarterly Report on General Household Survey. The average median monthly wage in 2020 was calculated based on the average of the Q1, Q2, Q3, and Q4 reports.

<https://www.censtatd.gov.hk/en/data/stat_report/product/B1050001/att/B10500012020QQ01B0100.pdf>

<https://www.censtatd.gov.hk/en/data/stat_report/product/B1050001/att/B10500012020QQ02B0100.pdf>

<https://www.censtatd.gov.hk/en/data/stat_report/product/B1050001/att/B10500012021QQ03B0100.pdf>

<https://www.censtatd.gov.hk/en/data/stat_report/product/B1050001/att/B10500012020QQ04B0100.pdf>

^d^ The Ministry of Labor. Labor and Economic Indicators. <https://statdb.mol.gov.tw/html/mon/c1010.htm>

^e^ The Ministry of Health, Labor and Welfare. Wage Structure Basic Survey 2020. <https://www.mhlw.go.jp/toukei/itiran/roudou/chingin/kouzou/z2020/dl/01.pdf>

^f^ The Ministry of Employment and Labor. Survey Report on Labor Conditions by Employment Type. <https://www.index.go.kr/unify/idx-info.do?idxCd=8085>

^g^ The Ministry of Manpower. The Report on Labour Force in Singapore 2020. Nominal Median Monthly Income. <https://stats.mom.gov.sg/Pages/Labour-Force-In-Singapore-2020.aspx>

^h^ Data for mainland China, Hong Kong, Japan, South Korea and Singapore are from the World Bank (2021). <https://data.worldbank.org/indicator/SP.POP.TOTL?locations=CN-HK-JP-KR-SG&name_desc=true>

Data for Taiwan is from the Ministry of the Interior, 2021 Demographic Fact Book Republic of China. <https://www.ris.gov.tw/app/portal/346>

^i^ Central Intelligence Agency. The World Factbook. 65 years and over (2023 est.). <https://www.cia.gov/the-world-factbook/>

Life expectancy at birth (2023 est.). <https://www.cia.gov/the-world-factbook/>

^j^ United Nations. (2019). World mortality 2019 highlights. <https://www.un.org/development/desa/pd/content/world-mortality-2019-highlights>

^k^ The World Bank.

^l^ Health Bureau. Domestic Health Accounts 2019/20. <https://www.healthbureau.gov.hk/statistics/en/dha/dha_summary_report.htm>

^m^ Ministry of Health and Welfare. 2020 National Health Expenditure. <https://www.mohw.gov.tw/lp-130-2.html> Out-of-pocket payment per capita for Taiwan was converted to USD using 2019 PPP exchange rates from <https://data.nasdaq.com/data/ODA/TWN_PPPEX-taiwan-province-of-china-implied-ppp-conversion-rate-lcu-per-usd>

^n^ Health Bureau. <https://www.healthbureau.gov.hk/statistics/en/health_statistics.htm>

^o^ Ministry of Health and Welfare. Statistics Of Medical Care Institution & Hospital Utilization 2021. <https://www.mohw.gov.tw/cp-6528-70693-2.html>

^p^ National Health Commission. Statistical bulletin of Chinese health development 2020. <http://www.nhc.gov.cn/guihuaxxs/s10743/202107/af8a9c98453c4d9593e07895ae0493c8.shtml>

^q^ Hospital Authority. Hospital Authority Statistical Report 2020-2021. Outpatient visits were calculated based on healthcare facilities managed by the Hospital Authority. Average hospital length of stay was calculated based on healthcare facilities managed by the Hospital Authority. <https://www3.ha.org.hk/data/HAStatistics/DownloadReport/8?isPreview=False>

^r^ Ministry of Health and Welfare. National Health Insurance Annual Statistical Report 2020. Outpatient and Inpatient Medical Benefit Claims by Ownership, (pp. 348–349). Outpatient visits per capita was calculated using total outpatient cases divided by total population in the society. <https://dep.mohw.gov.tw/dos/lp-5103-113-xCat-y109.html>

^s^ Ministry of Health, Labour and Welfare. Medical benefit survey 2020. Table 1. Outpatient visits per capita was calculated using all outpatient cases divided by total population. Hospital length of stay was calculated using the sum of length of stay of inpatients divided by total number of inpatients. <https://www.e-stat.go.jp/stat-search/files?page=1&toukei=00450389&tstat=000001044924>

^t^ National Health Insurance Service. National Health Insurance Statistical Yearbook. Service Benefits by Type of Provider (Total). Outpatient visits per capita was calculated using all outpatient visits divided by total population (attendances to pharmacy were excluded). Hospital length of stay was calculated using total inpatient days divided by total number of inpatients.

<https://kosis.kr/statHtml/statHtml.do?orgId=350&tblId=TX_35001_A037&language=en&conn_path=I3>

^u^ Ministry of Health. Hospital Admissions and Public Sector Outpatient Attendances, Annual. Outpatient visits were calculated based on public sector outpatient attendances (including dentists).

[https://data.gov.sg/dataset/hospital-admissions-and-public-sector-outpatient-attendances-annual?view_id=81b22932-9895-4efa-8615-df22823e4173&resource_id=ba3c89a7-cfc2-4c87-afe3-b688b0f0ad75](https://beta.data.gov.sg/datasets/d_a5267c58f60b20f8e04576261abfac93/view)

^v^ SingHealth Duke-NUS Academic Medical Centre. Singhealth Group Overall Key Figures and Statistics. Hospital length of stay was calculated using total inpatient days divided by total number of inpatients (including acute care, polyclinics, and community hospitals).

[https://www.singhealth.com.sg/about-singhealth/newsroom/Documents/SingHealth Duke-NUS AR1920-OVERVIEW_final.pdf](https://www.singhealth.com.sg/about-singhealth/newsroom/Documents/SingHealth%20Duke-NUS%20AR1920-OVERVIEW_final.pdf)

**Appendix B**

We use another two widely used measurements of capacity to pay: 40% of total income subtracting subsistence expenditures on food (Xu et al., 2003) and 40% of total income subtracting subsistence expenditures on food, rent, and utilities (Thomson et al., 2019); (2) Our lung cancer cost estimation is affected by varying hospitalization durations for patients in a society. A longer hospitalization stay can be observed in Korea (see Appendix Table A1). In the alternative specification, we calculate lung cancer costs using the average lung cancer hospitalization stay in OECD and EU countries for all East Asian societies. Results are presented in Figures B1 and B2.

**Figure B1.** CHS of lung cancer using alternative measures of capacity to pay


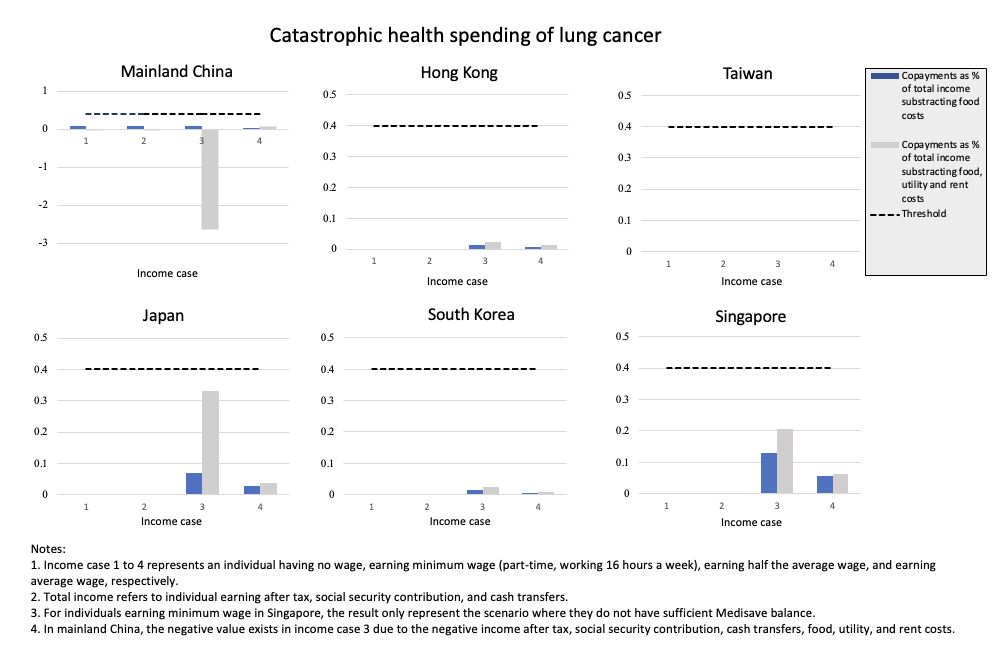


**Figure B2.** CHS of lung cancer based on OECD countries’ and European countries’ average length of stay of lung cancer patients (8.47 days and 8.77 days, respectively)


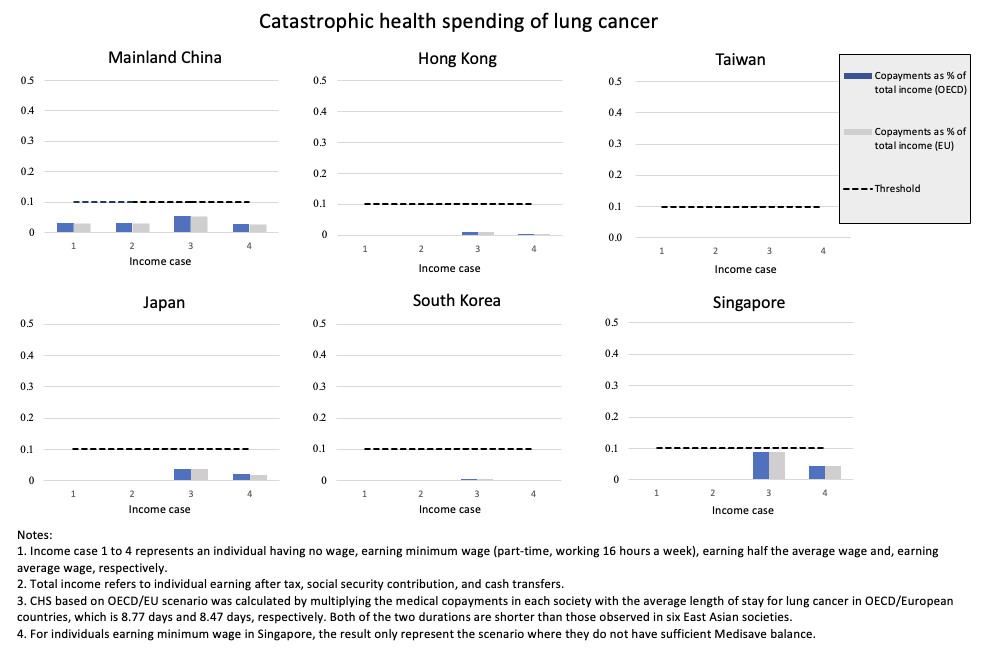


Data source.

OECD Health Statistics. Hospital average length of stay for malignant neoplasm of trachea, bronchus, and lung. <https://stats.oecd.org/Index.aspx?ThemeTreeId=9>

Eurostat. In-patient average length of stay for malignant neoplasm of trachea, bronchus, and lung. <https://ec.europa.eu/eurostat/databrowser/view/HLTH_CO_INPST__custom_1857895/default/table>

**Appendix C**

**Table C1.** Outpatient visits per person and the average length of stay before COVID-19 in six societies

|  | Outpatient visits per person (visits) | | | Average length of stay (days) | | |
| --- | --- | --- | --- | --- | --- | --- |
|  | 2018 | 2019 | 2020 | 2018 | 2019 | 2020 |
| Mainland China^a^ | 6 | 6.2 | 5.5 | 9.3 | 9.1 | 8.5 |
| Hong Kong^b^ | 2.65 | 2.47 | 2.35 | 7.2 | 7.5 | 8 |
| Taiwan^c^ | 15.26 | 15.57 | 14.44 | 9.39 | 9.29 | 9.35 |
| Japan^d^ | 9.00 | 9.3 | 8.88 | 15.26 | 15 | 14.5 |
| South Korea^e^ | 17.55 | 17.84 | 15.41 | 19.87 | 19.71 | 20.9 |
| Singapore | 2.46^f^ | 2.54^f^ | 2.18^f^ | 4.8^g^ | 4.9^g^ | 4.8^g^ |

Data sources.

^a^ National Health Commission. China Health Statistical Yearbook 2019/2020/2021.

^b^ Hospital Authority. Hospital Authority Statistical Report 2018-2019/2019-2020/2020-2021.

^c^ Ministry of Health and Welfare. National Health Insurance Annual Statistical Report 2018/2019/2020.

^d^ Ministry of Health, Labour and Welfare. Medical benefit survey 2018/2019/2020.

^e^ National Health Insurance Service. National Health Insurance Statistical Yearbook 2018/2019/2020.

^f^ Ministry of Health. Hospital Admissions and Public Sector Outpatient Attendances, Annual.

^g^ SingHealth Duke-NUS Academic Medical Centre. Singhealth Group Overall Key Figures and Statistics 2018/2019/2020.

**Appendix D**

**D1. Data sources for Table 1**

^a^ The People’s Government of Beijing Municipality. (2021). Reimbursement rules of Urban Employee Basic Medical Insurance.

<https://www.beijing.gov.cn/fuwu/bmfw/bmdh/sb/ylbx/bs/dyjf/202109/t20210910_2490612.html>

Beijing Municipal Human Resources and Social Security Bureau. (2017). Rules for the Implementation of Beijing Urban and Rural Resident Medical Insurance.<http://ybj.beijing.gov.cn/zwgk/2020_zcwj/202002/t20200211_1627784.html>

^b^ Beijing Municipal Civil Affairs Bureau. (2014). Notice on Adjustment of Medical Assistance Standards. <http://www.beijing.gov.cn/zhengce/zhengcefagui/qtwj/201912/t20191219_1324503.html>

^c^ Minimum wage of Beijing is derived from <http://xiaofei.people.com.cn/n1/2020/0715/c425315-31783666.html>

^d^ Beijing Municipal Civil Affairs Bureau. (2017). Notice on Adjustment of Medical Assistance Standards for Social Assistance Recipients. <http://www.bdpf.org.cn/n1508/n1509/n1513/n2953/c68602/content.html>

For people in extreme poverty (特困供养人员), the assistance rate of MFA is 100%.

^e^ Leung et al., 2017. <https://doi.org/10.1016/b978-0-12-803678-5.00068-0>

^f^ Hospital Authority. Mechanism of Waiving of Medical Charges. The target population are the recipients of Comprehensive Social Security Assistance (CSSA), Level 0 voucher holders of the Pilot Scheme on Residential Care Service for the Elderly, Old Age Living Allowance (OALA) recipients aged 75 or above, the low-income group, chronically ill patients, and elderly patients who have little income or assets.

<https://www.ha.org.hk/visitor/ha_visitor_index.asp?Parent_ID=10044&Content_ID=10047&Ver=HTML>

^g^ The copayment rate for outpatients was estimated using medical copayments divided by total medical points. Data are derived from National Health Insurance Annual Statistical Report 2020 (p. 346). <https://dep.mohw.gov.tw/dos/lp-5103-113-xCat-y109.html>

^h^ National Health Insurance Administration, Ministry of Health and Welfare. Copayments. <https://www.nhi.gov.tw/Content_List.aspx?n=BCB1A5D2CBACD6E0&topn=5FE8C9FEAE863B46>

^i^ National Health Insurance Administration, Ministry of Health and Welfare. National Health Insurance Act. Copayments are exempted for patients with severe illness.

<https://law.moj.gov.tw/ENG/LawClass/LawAll.aspx?pcode=L0060001>

^j^ Department of Social Welfare, Taipei City Government. Medical subsidies.

<https://dosw.gov.taipei/Content_List.aspx?n=CBF8AAEF232010B8>

Ministry of Health and Welfare. County (City) Regulations for subsidizing medical treatment.

<https://law.moj.gov.tw/LawClass/LawAll.aspx?pcode=D0050082>

Taiwan’s city and county governments also provide MFA to low- and middle-low-income elderly people. References are as follows:

Hsinchu County Government, Department of Social Affairs. Medical assistance for lower-income elderly participating in National Health Insurance who cannot afford medical expenses. <https://society.hccg.gov.tw/ch/home.jsp?id=119&parentpath=0,3,25&mcustomize=onemessages_view.jsp&toolsflag=Y&dataserno=201603290036&t=SocietyOnes&mserno=201603090025>

Ministry of Health and Welfare.1957 welfare consultation hotline. <https://1957.mohw.gov.tw/servlet/QACtrl?func=QAView&sid=12871>

^k^ Minimum living standard, low-income line, and middle-to-low-income line in Taiwan are from the Ministry of Health and Welfare.

<https://dep.mohw.gov.tw/DOSAASW/cp-566-49605-103.html>

<https://law.moj.gov.tw/LawClass/LawAll.aspx?pcode=D0050082>

^l^ Ministry of Health, Labour and Welfare. Copayments.

<https://www.mhlw.go.jp/file/06-Seisakujouhou-12400000-Hokenkyoku/0000209890.pdf>

For children who are aged 0-15 and meet eligibility criteria, medical copayment is totally waived. <https://kosodate.city.hachioji.tokyo.jp/soshiki/kosodateshienka/kosodateshienka_shomu_kyufu_jidoteatenyukotanto_hitorioyatanto/256.html>

<https://www.city.ota.tokyo.jp/seikatsu/kodomo/teate/kodomonyuui.html>

^m^ Ministry of Health, Labour and Welfare. 高額療養費制度を利用される皆さまへ. <https://www.mhlw.go.jp/stf/seisakunitsuite/bunya/kenkou_iryou/iryouhoken/juuyou/kougakuiryou/index.html>

^n^ Ministry of Health, Labour and Welfare. Social Welfare. Outline of the Public Assistance System.

<https://www.mhlw.go.jp/english/policy/care-welfare/social-welfare/index.html>

^o^ Minimum wage in Tokyo is 1,013 Yen hourly and 174,236 Yen (1013*40*4.3) monthly in 2020. Data is from the Ministry of Health, Labour and Welfare of Japan. <https://www.mhlw.go.jp/stf/seisakunitsuite/bunya/koyou_roudou/roudoukijun/minimumichiran/>

^p^ National Health Insurance Service. Insurance Benefits. <https://www.nhis.or.kr/english/wbheaa02600m01.do>

^q^ Ministry of Health and Welfare. Medical Aid Act. [https://www.law.go.kr/LSW/LsiJoLinkP.do?lsNm=%EC%9D%98%EB%A3%8C%EA%B8%89%EC%97%AC%EB%B2%95&paras=1&docType=JO&languageType=KO&joNo=000300000#](https://www.law.go.kr/LSW/LsiJoLinkP.do?lsNm=%EC%9D%98%EB%A3%8C%EA%B8%89%EC%97%AC%EB%B2%95&paras=1&docType=JO&languageType=KO&joNo=000300000)

^r^ Ministry of Health and Welfare. <https://www.mohw.go.kr/react/policy/index.jsp?PAR_MENU_ID=06&MENU_ID=06350103&PAGE=3&topTitle=%E5%8F%97%E7%9B%8A%E4%BA%BA%E9%80%89%E6%8B%A9%E6%A0%87%E5%87%86>

^s^ The standard median income for a 1-person family is 1,757,194 Won in 2020. Data is from the Ministry of Health and Welfare of Korea.

<https://www.mohw.go.kr/react/policy/index.jsp?PAR_MENU_ID=06&MENU_ID=06350109&PAGE=9&topTitle=%E6%A0%87%E5%87%86%E6%94%B6%E5%85%A5%E4%B8%AD%E4%BD%8D%E6%95%B0>

^t^ Ministry of Health and Welfare. 2018 Welfare Services Guidelines.

<https://www.mohw.go.kr/eng/nw/nw0103.jsp?PAR_MENU_ID=1007&MENU_ID=100704>

^u^ Ministry of Health. Medishield Life. Medishield Life is a basic medical insurance scheme to help Singaporeans cope with large hospital bills and selected costly outpatient treatments. Medisave pay the most of outpatient bills. <https://www.moh.gov.sg/cost-financing/healthcare-schemes-subsidies/medishield-life>

^v^Ministry of Health. Medifund. <https://www.moh.gov.sg/cost-financing/healthcare-schemes-subsidies/medifund>

Medifund Silver and Medifund Junior provide assistance for the needy old adults and children. Two programs tend to provide more generous benefits. References are as follows:
Ministry of Health. <https://www.moh.gov.sg/newsroom/medifund-continues-to-assist-needy-singaporeans-with-their-healthcare-needs_2021>

Ministry of Finance. <https://www.mof.gov.sg/news-publications/forum-replies/Government-spending-must-be-sustainable-and-not-burden-future-generations>

^w^ As there is no public income-based eligibility in Singapore, we used the threshold ComCare Short-to-Medium Term Assistance: household income of $1,900 a month or per capita income of $650 a month (Ministry of Social and Family Development, 2017). This threshold is consistent with the poverty line estimated by scholars at $1,913 for a household (Ng, Y. H. I., 2020).

**D2. Data sources for Table 2**

^a^ National Healthcare Security Administration. Statistical Communique on the Development of the National Medical Security Service 2020. <http://www.nhsa.gov.cn/art/2021/6/8/art_7_5232.html>

b National Health Commission. China Health Statistical Yearbook 2021. <http://www.nhc.gov.cn/mohwsbwstjxxzx/tjtjnj/202305/304a301bfdb444afbf94b1a6c7f83bca/files/2ac5438bcec3473686d9fb3c9d2187f3.pdf>

^c^ Beijing Municipal Civil Affairs Bureau. (2017). Notice on Adjustment of Medical Assistance Standards for Social Assistance Recipients. <http://www.bdpf.org.cn/n1508/n1509/n1513/n2953/c68602/content.html>

^d^ Hospital Authority. <https://www.legco.gov.hk/yr20-21/chinese/fc/fc/w_q/fhb-h-c.pdf> (p. 166)

e Hospital Authority. Fees and Charges. <https://www.ha.org.hk/visitor/ha_visitor_index.asp?Content_ID=10045&Lang=ENG&Dimension=100&Parent_ID=10044&Ver=HTML>

^f^ We referred to inpatient bed-days of respiratory tract cancer in 2005, published in Chan-Yeung et al., 2008. The burden of lung disease in Hong Kong: A report from the Hong Kong Thoracic Society. *Respirology (Carlton, Vic.), 13 Suppl 4*, S133–S165. <https://doi.org/10.1111/j.1440-1843.2008.01394.x>

^g^ Hospital Authority. Mechanism of Waiving of Medical Charges. <https://www.ha.org.hk/visitor/ha_visitor_index.asp?Parent_ID=10044&Content_ID=10047&Ver=HTML>

^h^ Total recipients were estimated by the number of total low-income people and middle-to-low-income people. Data are from <https://dep.mohw.gov.tw/DOS/cp-5337-62357-113.html>

According to Taiwan’s regulation for medical subsidies, MFA covers low-income, middle-to-low-income people, and people who have severe injuries and illnesses with income not exceeding 150% of the minimum living standard. Apart from MFA, Taiwan set a medical copayment waiving mechanism for the eligible population or treatment such as severe illness, child delivery, low-income population, veterans, and centenarians. People for whom total medical copayment was waived accounted for 15.18% in 2020.

<https://law.moj.gov.tw/LawClass/LawAll.aspx?pcode=D0050082>

<https://www.nhi.gov.tw/Content_List.aspx?n=E12BF2F395F122B0&topn=5FE8C9FEAE863B46>

i Ministry of Health and Welfare. National Health Insurance Annual Statistical Report 2020. (p. 756). <https://dep.mohw.gov.tw/dos/lp-5103-113-xCat-y109.html>

^j^ Ministry of Health and Welfare.<https://www.mohw.gov.tw/dl-37791-18e9acb4-e8d2-432c-a857-3e153dad0e71.html>

^k^ Department of Social Welfare, Taipei City Government. Medical financial assistance. <https://dosw.gov.taipei/Content_List.aspx?n=CBF8AAEF232010B8>

^l^ Ministry of Health, Labour and Welfare. Handbook of Health and Welfare Statistics. Table 3-5. Actual number and ratio of public assistance recipients by type of assistance, by fiscal year 2020. <https://www.mhlw.go.jp/english/database/db-hh/3-1.html>

m Ministry of Health, Labour and Welfare. Medical benefit survey 2020. Table 1.

<https://www.e-stat.go.jp/stat-search/files?page=1&layout=datalist&toukei=00450389&tstat=000001044924&cycle=0&tclass1=000001044945&tclass2=000001171146&cycle_facet=tclass1%3Acycle&tclass3val=0&metadata=1&data=1>

^n^ Ministry of Health, Labour and Welfare. Medical benefit survey 2020. Table 3.

https://www.e-stat.go.jp/stat-search/files?page=1&layout=datalist&toukei=00450389&tstat=000001044924&cycle=0&tclass1=000001044945&tclass2=000001171146&stat_infid=000032251633&cycle_facet=tclass1%3Acycle&tclass3val=0&metadata=1&data=1

^o^ Ministry of Health, Labour and Welfare. Social Welfare. Outline of the Public Assistance System. <https://www.mhlw.go.jp/english/policy/care-welfare/social-welfare/index.html>

^p^ National Health Insurance Service, National Health Insurance Statistical Yearbook. <https://kosis.kr/statHtml/statHtml.do?orgId=350&tblId=TX_35001_A001&language=en&conn_path=I3>

The recipients of Medical Aid include Basic Livelihood Security Program (BLSP) beneficiaries, other vulnerable populations (i.e., disaster victims, adopted children aged 18 or below), and meritorious citizens such as the holders of national intangible cultural heritage, individuals who have been injured, or family members of those who died while helping others.

q National Health Insurance Service. National Health Insurance Statistical Yearbook. Service Benefits by Type of Provider (Total).

<https://kosis.kr/statHtml/statHtml.do?orgId=350&tblId=TX_35001_A037&language=en&conn_path=I3>

^r^ OECD. Hospital average length of stay by diagnostic categories. <https://stats.oecd.org/index.aspx?DataSetCode=HEALTH_proc>

^s^ As MFA is a parallel program to the SHI scheme, the assistance rate in Korea is estimated using average charged copayments for MFA recipients relative to copayments for non-recipients.

Data source: Ministry of Health and Welfare. 2018 Welfare Services Guidelines. <https://www.mohw.go.kr/eng/nw/nw0103.jsp?PAR_MENU_ID=1007&MENU_ID=100704>

^t^ Ministry of Health. [https://www.moh.gov.sg/news-highlights/details/medifund-continues-to-assist-needy-singaporeans-with-their-healthcare-needs-2020 - :~:text=1.,2019 to 31 March 2020.](https://www.moh.gov.sg/news-highlights/details/medifund-continues-to-assist-needy-singaporeans-with-their-healthcare-needs-2020#:~:text=1.,2019%20to%2031%20March%202020.)

u Outpatient copayments in Singapore and were averaged based on the unit cost for consultation, accident & emergency, dental and day surgery in hospitals, and polyclinics in the public sector.

<https://www.skh.com.sg/patient-care/outpatient-consultation-charges>

<https://www.sgh.com.sg/patient-care/visiting-specialist/charges-payments-singapore-general-hospital>

<https://www.ktph.com.sg/patients/hospital-charges>

<https://www.ttsh.com.sg/Patients-and-Visitors/Your-Clinic-Visit/Pages/Outpatient-Charges.aspx>

[https://www.ah.com.sg/Pages/For Patients/Charges--Payment.aspx](https://www.ah.com.sg/Pages/For%20Patients/Charges--Payment.aspx)

<https://www.nuh.com.sg/patients-visitors/Pages/Charges-Payment.aspx>

<https://www.ntfgh.com.sg/for-patients-and-visitors/Pages/Charges-and-Payment.aspx>

<https://polyclinic.singhealth.com.sg/patient-care/charges-payment>

v Inpatient costs before Medishield claims were the total bill amount averaged based on all common diseases (ward b2 and c).

<https://www.moh.gov.sg/cost-financing/fee-benchmarks-and-bill-amount-information/BodyPartsSearch>

^w^ National University Cancer Institute Singapore. Estimated based on the average length of stay for lungs and abnormal growth with very severe complications in the public sector.

<https://www.ncis.com.sg/For-Patients-and-Visitors/Pages/Total-Bill-Estimates-and-Benchmarks.aspx>

^x^ According to the Medical Endowment Scheme Annual Report 2020/2021, nearly 90% of the applications received full assistance from Medifund. Therefore, we consider the assistance rate for outpatient, inpatient, and severe illness to be 100%, acknowledging that a small proportion of patients may receive partial assistance due to their family financial status or other special reasons. The information is derived from: Medical Endowment Scheme Annual Report 2020/2021. Exhibit 7. Percentage of Medifund-assisted Admissions/Attendances/6-Bed-Months that received Full Assistance from Medifund and Medifund Silver in FY19 and FY20. <https://www.moh.gov.sg/docs/librariesprovider5/pressroom/press-releases/medical-endowment-scheme-annual-report-2020-2021.pdf>
